# microRNAs as reference genes for quantitative PCR in cotton

**DOI:** 10.1371/journal.pone.0174722

**Published:** 2017-04-17

**Authors:** Anna Karoline Silva Fausto, Tatiane da Franca Silva, Elisson Romanel, Maite F. S. Vaslin

**Affiliations:** 1 Lab. Virologia Molecular Vegetal, Depto. Virologia, IMPPG, Universidade Federal do Rio de Janeiro, UFRJ, Rio de Janeiro, RJ, Brasil; 2 Departamento de Biotecnologia, Escola de Engenharia de Lorena (EEL), Universidade de São Paulo (USP), Lorena, SP, Brasil; Universidade de Lisboa Instituto Superior de Agronomia, PORTUGAL

## Abstract

Cotton (*Gossypium hirsutum*) is the most important non-food plant in the world. Studies concerning the fiber quality and plant fitness of cotton at molecular level depend on high sensitive and reproducible gene-expression assays. However, only a few reports have described genes suitable for normalizing gene expression data. In this study, we report for the first time that microRNAs (miRNAs) are reliable reference genes (RGs) for cotton gene expression normalization in quantitative real-time reverse transcription (RT)-PCR. The stability of cotton miRNAs was assayed in root, stem, leaf and flower samples from three different cultivars [FiberMax (FM966), Delta Opal (DO) and Cedro] and under conditions of biotic stress caused by infection with Cotton leafroll dwarf virus (CLRDV). The stability of mRNAs already described as reference genes in cotton was also assessed. The geNorm, NormFinder, BestKeeper and ΔCt algorithms were used to select the best reference genes. In 8 of the 12 sets tested, miRNAs (miR172, 168 and 390) were found to be the best RGs. To validate the best selected RGs, miR159, miR164, miR2118, miR2910, miR3476, *GhDCL2* and *GhDCL4* expression levels were evaluated under biotic stress conditions, and miR164 and a putative *myo-inositol oxigenase* gene (*GhMIOX*) were assessed in leaves and flowers. The RGs selected in this work proved to be excellent reference genes in the two cases studied. Our results support the use of miRNAs as reference genes for miRNA and protein-coding genes.

## Introduction

Cotton is one of the most important crop in the world. It is commercially grown in the temperate and tropical regions of more than 60 countries and represents the main source of renewable textile fibers. Moreover, cotton seed is an important source of oil, and its use as a fuel is currently growing. The allotetraploid upland cotton (AD)_1_ (*Gossypium hirsutum* L.) represents more than 90% of cultivated cotton worldwide. Based on the importance of cotton and the high demand for agricultural sources as pesticides and others to achieve high production levels, there is a steady need for the biotechnological improvement of this crop.

In this context, gene expression studies represent a crucial tool for understanding at the molecular level biologically important processes in cotton, such as fiber development and biotic and abiotic stress responses, among others, particularly now because the cotton genome has been made public [1–3].

Quantitative real-time reverse transcription polymerase chain reaction (qRT-PCR) or quantitative polymerase chain reaction (qPCR) is a powerful technique for the study of gene expression due to its high sensitivity, good reproducibility and ability to quantify expression over a wide range, compared to other experimental techniques used for the same purpose, such as northern blot, microarray and RNA-seq analyses [4]. Even in comparison with the emerging digital PCR (dPCR) technique, which allows for the precise and absolute quantification of small-percentage differences in rare nucleic acids, qPCR has been shown to be more versatile and precise by more than 9 orders of magnitude [5].

Despite its numerous advantages, qPCR requires the endogenous normalization of the data to achieve a correct interpretation of gene expression. This normalization is required to reduce mistakes due to the variability in the RNA quality between samples, the presence of co-purified inhibitors, different efficiencies of the reverse transcription (RT) and PCR steps among samples, and RNA degradation [6,7]. Therefore, normalization is essential to correct many variations that may affect the quantification of results and has been the most important challenge of the qPCR technique [8,9]. Normalization is performed using genes with invariant abundance levels under distinct experimental conditions. Genes used for this purpose are called reference genes (RGs). As a consensus, it is assumed that such genes have constant expression levels in the different tissues or cells analyzed and that their expression does not affect the experimental parameters [10]. Initially, *ribosomal small subunit* (*18S rRNA*), *elongation factor 1α* (*EF1α*), *actin* (*ACT*), *glyceraldehyde-3-phosphate dehydrogenase* (*GAPDH*), *ubiquitin* (*UBQ*) and/or *UBI1*, among others, were used as reference genes due to their basic functions in cells. However, several reports have shown that these genes may vary considerably under several experimental conditions [11–13]. For each experimental condition, the selection of appropriate RGs is thus essential to avoid a poor or inappropriate expression analysis [9].

Recently, small non-coding RNAs, especially microRNAs (miRNAs), have been investigated as important regulators of gene expression in mammalian cells and plant tissues [14–18]. miRNAs are involved in the regulation of plant development, signal transduction, the expression of transcription factors and protein degradation and participate in the response to biotic and abiotic stresses [19]. With advances in second-generation large-scale sequencing techniques, a large quantity of new genes and miRNAs have been identified. To understand the pathways regulated by these miRNAs, using qPCR assays to amplify miRNAs is becoming routine and has helped decipher miRNA functions in several commercial plant species, such as sugarcane and cotton under conditions of abiotic stress [20–22] and tobacco and cotton under conditions of biotic stress [23–25]. In most of the previous miRNA expression profile analyses performed in plants, protein-coding genes were used as RGs.

miRNA expression profile studies in humans and plants have shown that the expression of miRNAs may be more stable than that of coding RNAs [26–29]. These authors propose that miRNAs should be used as RGs in studies of protein-coding genes and especially in studies of miRNA expression. Moreover, a recent study argues that it is better to normalize target genes with RGs of the same class to avoid problems due to distinct biogenesis [30]. Plant researchers have begun to use miRNA normalization in qPCR assays in soybean plants [28]. Due to the high stability of miRNAs, a few studies have already reported their use for protein-coding mRNA and/or miRNA expression studies, including those in citrus [31], wheat [26], longan [32], lettuce [33] and, more recently, *Brassica napus* [30] and barley [34].

Artico and collaborators (2010) [35] have identified the protein-coding genes *GhUBQ14* (poly-ubiquitin) and *GhPP2A1* (protein phosphatase 2A catalytic subunit) as RGs for the normalization of gene expression in different cotton plant organs, and *GhACT4* (actin 4) and *GhUBQ14* and *GhMZA* (clathrin adaptor complexes medium subunit family protein) and *GhPTB* (polypyrimidine tract-binding protein homolog) for flower and fruit development, respectively. Protein-coding RGs were also selected for the study of gene expression under abiotic stress conditions in cotton [36]. However, to date, no study has been performed to identify protein-coding genes or miRNA, to use as reference genes in cotton under biotic stress conditions.

In the present study, miRNA reference genes were identified as RGs for protein-coding and miRNA gene expression studies for three commercially important cotton (*G*. *hirsutum*) varieties. To the best of our knowledge, this is the first report on the use of miRNAs as RGs for cotton. miRNAs and protein-coding genes were selected based on their stability in the root, stem, leaf and flower tissues of the cotton varieties Delta Opal (DO, which is planted worldwide), FiberMax 966 (FM966) and BRS Cedro (largely planted in Brazil). A set of RG miRNAs was also selected for the biotic stress studies, using the viral cotton blue disease, caused by Cotton leafroll dwarf virus, as a model. The approaches used to validate the best RG selected revealed that the miRNAs were the best reference genes not only for miRNA but also for protein-coding genes.

## Methods

### Plant material

Three cotton (*Gossypium hirsutum* L.) varieties were chosen based on their importance for Brazilian cotton growers: FiberMax966 (Aventis Crop Science) from Australia, Delta Opal (Delta and Pine Land Co.) and BRS Cedro, developed by EMBRAPA (Empresa Brasileira de Pesquisa Agropecuária, Brazil). Delta Opal (DO) and FiberMax966 (FM) are elite varieties grown worldwide. Plants were grown under controlled temperature (25 +/- 2°C) under natural photoperiod in a greenhouse at the Universidade Federal do Rio de Janeiro (UFRJ). Two biological independent replicates of leaves, shoots and roots of one month old plants from the three varieties were harvested. Flowers of FM966 and Delta Opal with 0 dpa (days post anthesis) and Cedro flower buds with 10 mm diameter were also harvested. All samples were immediately frozen in liquid N_2_ and stored at -80°C until total RNA extraction.

### Plant virus infection

Cotton (*Gossypium hirsutum*) plants of the cultivar FM966 (Fibermax966), susceptible to Cotton blue disease, were grown in green house conditions (26 +/-2°C) and at 30 days pos germination were infected with CLRDV using viruliferous aphids (*Aphis gossypii* Glover) as described previously [37]. Leaves of non-infected plants were used as non-infected control. Two biological replicates were analyzed for treatment.

### RNA isolation and cDNA synthesis

Frozen samples were ground to a fine powder in liquid nitrogen. Total RNAs were extracted from 100 mg of each macerated plant tissue using the Invisorb Spin Plant RNA Mini Kit (Stratec Medical) as described by the manufacturer. RNA concentration and purity (A_260/230_ and A_260/280_) were determined using a NanoDrop™ spectrophotometer ND-1000 (Thermo Scientific). To avoid DNA contamination, samples were treated with RNAse-free DNAseI (Fermentas Co.). cDNA synthesis was performed by multiplex technique [38], adding a pool of 50 mM each of the three gene-specific stem–loop primers, design as described previously [20], and including oligonucleotide dT (Invitrogen), plus 200 ng total RNA. cDNA synthesis was performed with RevertAid First Strand cDNA Synthesis Kit (Thermo Scientific) following manufacturer’s instructions. Finally, to verify the absence of gDNA in the RNA samples, a PCR was performed using 200 ng of DNAse-treated RNA as template and primers for the *GhXTH* gene (*Xyloglucan endotransglucosylase*/*hydrolase*). To amplify *GhXTH* partial sequence, the previously described primers XTH for (5’—GGAAAGGGTGACAGGGAACA– 3’) and XTH rev (5’–GGCTGGAGTTTTGGGTATGG– 3’) were used [39]. All cDNA samples were 25-fold diluted with RNase-free water before use as a template in the qPCR analysis.

### Selection of reference genes and primer design

Six cotton miRNAs were selected as putative reference genes for qPCR analysis: miR159, miR166, miR167, miR168, miR172 and miR390. All of them show uniform and stable expression levels in leaves and during infection caused by CLRDV [37]. Four protein-coding reference genes already described for cotton were selected: *actin 4* (*GhACT*), *elongation factor 1-alfa 5* (*GhEF1*), *protein phosphatase 2A* (*GhPP2A1*) and *ubiquitin 14* (*GhUBQ14*) [35, 36]. In addition, *ribosomal 18S* (*Gh18S*) (*GHU42827*) was also selected based on previous studies in *Barley yellow dwarf virus* and *Cereal yellow dwarf virus* quantification in the resistant and susceptible wheat lines [40]. Blast search were done to obtain 18S primers in cotton. Table 1 and S1 Table show the primers pairs used in this work. Specific forward primers were designed for each miRNA based on the each cotton miRNA sequence and used together with the universal reverse primer for miRNA (5’ GTCGTATCCAGTGCAGGGTCCGAGGTATTCGCACTGGATACGACNNNNNNN3’) [38] for RT reactions, were N represent the primer specific portion. This universal primer contains a stem-loop structure consisting of 44 conserved and seven variable nucleotides that are specific to the 3’ end of the miRNA sequence.

**Table 1:** Oligonucleotides used in the qPCR assays for the candidate RGs. The amplicon characteristics and primer sequences used for the detection of Gh-miRNAs and mRNA using qPCR.

Gene abbreviation	GenBank accession	Symbol	Primer name	Primers sequences (5’-3’)	PCR efficiency	Regression coefficient (R^2^)	Amplicon (bp)	Reference
GhACT4	AY305726	ACT	GhACT4F	TTGCAGACCGTATGAGCAAG	0.94	0.986	200	[35]
			GhACT4R	ATCCTCCGATCCAGACACTG				
GhEF1a-5	DQ174254	EF	GhEF1a-5F	TCCCCATCTCTGGTTTTGAG	0.78	0.975	200	[35]
			GhEF1a-5R	CTTGGGCTCATTGATCTGGT				
GhPP2A1	DT545658	PP2A	GhPP2A1F	GATCCTTGTGGAGGAGTGGA	0.98	0.992	200	[35]
			GhPP2A1R	GCGAAACAGTTCGACGAGAT				
GhUBQ14	DW505546	UBQ	GhUBI14F	CAACGCTCCATCTTGTCCTT	0.85	0.999	200	[35]
			GhUBI14R	TGATCGTCTTTCCCGTAAGC				
Gh18S		18S	Gh18SF	CAGTCGGGGGCATTCGTA	0.95	0.990	200	[39]
			Gh18SR	CCCTGGTCGGCATCGTTTAT				
miR159ab		miR159	miR159F	TTTGGATTGAAGGGA	0.98	0.980	72	[37]
			miR159R*	GTTGGCTCTGGTGCAGGGTCCGAGGTATTCGCACCAGAGCCAACCCAGAGC				
miR166e		miR166	miR166F	GTCGGACCAGGCTTC	0.98	0.992	72	[37]
			miR166R*	GTTGGCTCTGGTGCAGGGTCCGAGGTATTCGCACCAGAGCCAACACGGGGA				
miR167g		miR167	miR167F	GCGGCGGGAAGCTGCCAGCAT	0.95	0.996	72	[37]
			miR167R*	GTTGGCTCTGGTGCAGGGTCCGAGGTATTCGCACCAGAGCCAACCCCAGAT				
miR168b		miR168	miR168F	GCGGCGGGTCGCTTGGTGCAGG	0.72	0.990	72	[37]
			miR168R*	GTTGGCTCTGGTGCAGGGTCCGAGGTATTCGCACCAGAGCCAACACTTCCC				
miR172f		miR172	miR172F	GCGGCGGTCTTGATGATGCTG	1.02	0.984	72	[37]
			miR172R*	GTTGGCTCTGGTGCAGGGTCCGAGGTATTCGCACCAGAGCCAACCGCCGAT				
miR390a		miR390	miR390aF	GCGGCGGGAAGCTCAGGAGGGA	0.90	0.978	72	[37]
			miR390aR*	GTTGGCTCTGGTGCAGGGTCCGAGGTATTCGCACCAGAGCCAACACGGCGC				

*- stem-loop RT-primers.

Before each specific reverse primer used for miRNA amplification a common sequence was inserted in the forward and reverser primers as Chen et al., 2005 [20].

### Quantitative real-time PCR

qRT–PCR was performed on an ABI 7500 Real-Time PCR System (Applied Biosystem) using SYBR Green I technology. Reactions were done with the Maxima™ SYBR Green/ROX qPCR Master Mix (Thermo Scientific) according to the manufacturer’s instructions. Real time PCR conditions were: 95°C for 10 min, hot-start incubation step, followed by 40 cycles of 95°C for 15 s, 60°C for 30 s, and 72°C for 35 s. Threshold and baselines were determined using the ABI 7500 Real-Time PCR System SDS Software v2.0. Two biological independent samples were analyzed for each set. All cDNA samples were amplified in technical triplicates starting with the same cDNA. A non-template control was also included. The absence of spurious amplification was confirmed by the presence of a single peak in the melting-curve analysis (S1 Fig).

### Data analysis

Primer efficiencies (E) were estimated for each experimental set by Miner software [41], that pin points the starting and ending points of PCR exponential phase from raw fluorescence data, and estimates primer set amplification efficiencies through a nonlinear regression algorithm without the need for a standard curve. Correlation coefficients (R^2^) were calculated using SDS software (ABI 7500 Real-Time PCR System v. 2.0) based on a standard curve generated using a twofold dilution series in duplicate over five dilution points. To calculate the expression stability of each of the 11 putative RG under different experimental conditions the following software tools were used: geNorm PLUS (trial version, Biogazelle, Zwijnaarde, Belgium) [42], NormFinder (ver. 0.953) [43], BestKeeper (ver. 1) [44] and comparative ΔCt method [45]. All software packages were used according to the manufacturer’s instructions. For geNorm and NormFinder the raw Cq values were transformed to quantities using the ΔCt method [43]. The highest relative quantities for each gene were set to 1.0. BestKeeper-1 and the comparative ΔCt method use raw Cq values.

### Validation of selected RGs

Validation of reference genes selected in this study was made using them as reference for cotton protein-coding genes and miRNAs which expression levels were previously studied. For that, the putative *myo-inositol oxygenase GhMIOX* (EST number 193219349 ortholog of *At4g26260*) [46] and miR164 [47] relative expression levels were assayed in flowers versus leaves. Relative expression of cotton *GhDCL2* and *4* [39], and of miR159, 164, 2118, 2910 and 3476 [37] were assayed in virus infected versus uninfected plants. All the primers used are described S1 Table. cDNAs were prepared as described above. Real-time PCRs were performed as described in quantitative real-time PCR section and primers annealing temperatures are shown in S1 Table.

The target genes were normalized using the best three miRNAs, three protein-coding genes and/or three protein-coding plus miRNAs HGK selected by GeNorm. The 2^-ΔΔCt^ method [45] was used to determine the expression levels. The parameters of two-tailed distribution and two samples assuming unequal variances were established. Means were considered significantly different when P < 0.05. Two biological samples, assayed in three technical replicates each, were evaluated for each experimental condition.

## Results

To select reliable miRNA RGs for cotton, six miRNA candidates (miRNA159ab, miR166e, miR167g, miR168b, miR172f and miR390a) were selected from the *G*. *hirsutum* NGS studies [37] and compared to five previously described cotton reference protein-coding genes: *ACT4*, *EF1α-5*, *PP2A1*, *UBQ14* [35] and *18S* (Table 1). Cotton Illumina small RNA libraries from uninfected and Cotton leafroll dwarf virus (CLRDV)-infected leaves [37] were used for the cotton miRNA selection. The six cotton miRNAs selected belong to different families in different functional classes based on Arabidopsis miRNA studies [48]. miRNA159 functions in the regulation of signaling pathways and development. miR166 is involved in leaf and vein development in Arabidopsis, while miR167 in involved in signaling pathways and flower development. miR168 plays a role in the biotic stress response and signal transduction during development; miR172 is involved in signaling, development and the stress response, and miR390 is involved in plant development.

The six cotton miRNA candidates, as well the five previously described cotton RGs, were evaluated for gene expression stability using qPCR assays of a set of 36 cotton samples grouped into 12 different experimental sets (Table 2). Experimental sets 1–4 were composed of plant organs: leaves (set 1), stems (set 2), roots (set 3) and flowers (set 4) of the three distinct cotton cultivars (cvs) FM966, DO and Cedro. Sets 5–7 were composed of combinations of leaves and stems (set 5), leaves and roots (set 6) and leaves and flowers (set 7). Sets 8–10 were composed of all organs of FM966 (set 8), DO (set 9) and Cedro (set 10). Finally, FM966 under biotic stress compose set 11 and all organs of the three cvs the set 12.

**Table 2:** Summary of three best candidate RGs in order of their expression stability, as calculated by geNorm, NormFinder, BestKeeper and ΔCt. The numbers 1, 2 and 3 represent the position of the candidates. n—number of samples analyzed.

Set	Samples		geNorm		NormFinder		BestKepper		ΔCt	
**1**	Leaves (n:6)	1	GhACT/GhPP2a	0.28	GhUBQ	0.24	miR172	0.12	miR168	1.27
		2			miR168	0.28	GhUBQ	0.59	GhUBQ	1.29
		3	GhEF1	0.49	miR172	0.35	miR167	0.68	GhEF1	1.29
**2**	Stems (n:6)	1	GhUBQ/GhACT	0.38	miR172	0.26	miR172	0.24	GhEF1	1.05
		2			GhEF1	0.26	GhEF1	0.34	miR172	1.09
		3	GhPP2a	0.43	miR390	0.39	miR159	0.49	GhPP2a	1.15
**3**	Roots (n:6)	1	GhUBQ/GhPP2a	0.26	GhEF1	0.30	miR172	0.11	GhEF1	1.28
		2			miR159	0.55	miR390	0.52	GhPP2a	1.30
		3	GhEF1	0.35	GhPP2a	0.59	miR159	0.76	GhUBQ	1.32
**4**	Flowers (n:6)	1	GhUBQ/GhACT	0.13	miR172	0.249	GhPP2a	0.26	miR172	0.94
		2			GhPP2a	0.369	miR172	0.28	GhPP2a	0.97
		3	GhPP2a	0.20	GhEF1	0.371	GhACT	0.32	GhEF1	1.01
**5**	Leaves and Stems (n:12)	1	Gh18S/GhPP2a	0.52	miR172	0.41	miR172	0.18	GhEF1	1.43
		2			miR168	0.45	miR159	0.61	miR168	1.47
		3	GhEF1	0.60	GhEF1	0.47	GhEF1	0.76	Gh18S	1.48
**6**	Leaves and roots (n:12)	1	miR168/Gh18S	0.62	miR172	0.36	miR172	0.16	GhEF1	1.57
		2			miR168	0.58	miR168	0.92	miR168	1.58
		3	GhPP2a	0.75	GhEF1	0.73	miR159	0.96	GhPP2a	1.60
**7**	Leaves and flowers (n:12)	1	GhACT/GhPP2a	0.48	GhUBQ	0.26	miR172	0.29	GhUBQ	1.46
		2			miR168	0.56	GhEF1	0.73	miR172	1.55
		3	GhEF1	0.56	miR172	0.6	GhACT	0.79	miR168	1.56
**8**	All organs/FM966 (n:8)	1	GhEF1/GhPP2a	0.29	miR168	0.19	miR172	0.29	miR172	1.47
		2			miR172	0.30	Gh18S	0.44	Gh18S	1.52
		3	Gh18S	0.53	miR390	0.35	GhEF1	0.58	miR168	1.55
**9**	All organs/Delta Opal (n:8)	1	miR168/miR172	0.39	miR168	0.19	miR172	0.28	miR168	1.56
		2			miR390	0.20	miR168	0.44	miR172	1.61
		3	miR390	0.47	miR172	0.32	GhEF1	0.56	miR390	1.66
**10**	All organs/Cedro	1	GhACT/GhPP2a	0.61	miR168	0.25	miR172	0.24	miR168	1.47
	(n:8)	2			GhPP2a	0.61	miR168	0.54	GhPP2a	1.5
		3	GhUBQ	0.71	GhUBQ	0.68	GhEF1	0.88	GhUBQ	1.58
**11**	Viral infection	1	miR168/GhPP2a	0.04	miR172	0.03	miR166	0.06	miR390	0.73
	(n:4)	2			GhACT	0.03	miR390	0.06	miR172	0.73
		3	miR390	0.09	miR390	0.05	miR172	0.08	miR168	0.73
**12**	All organs	1	GhEF1/GhPP2a	0.60	miR172	0.49	miR172	0.27	miR168	1.76
	(n:36)	2			miR168	0.60	GhEF1	0.77	miR172	1.76
		3	GhUBQ	0.77	GhEF1	1.11	miR168	0.92	GhEF1	1.8

High-quality total RNA from the different samples was isolated and reverse transcribed. After that, amplification efficiency for each primer pair of the 11 evaluated genes was determined in a qPCR assay using a ten-fold dilution series from a pooled cDNA template. The PCR efficiency values for all the candidate genes ranged from 0.72 for miR168 to 1.02 for miR172 (Table 1). The correlation coefficient (R^2^) ranged from 0.975 to 0.999, with *GhEF1a-5* and *GhUBQ14* at the low and high extremes, respectively (Table 1). Specific amplifications were confirmed by the occurrence of a single peak in the melting curve (S1A Fig) and by agarose gel electrophoresis, indicating a high PCR specificity for all the primer sets, which enabled all the candidate genes to be used in the next assay.

### Expression stability of miRNA candidate reference genes

**Fig 1:**
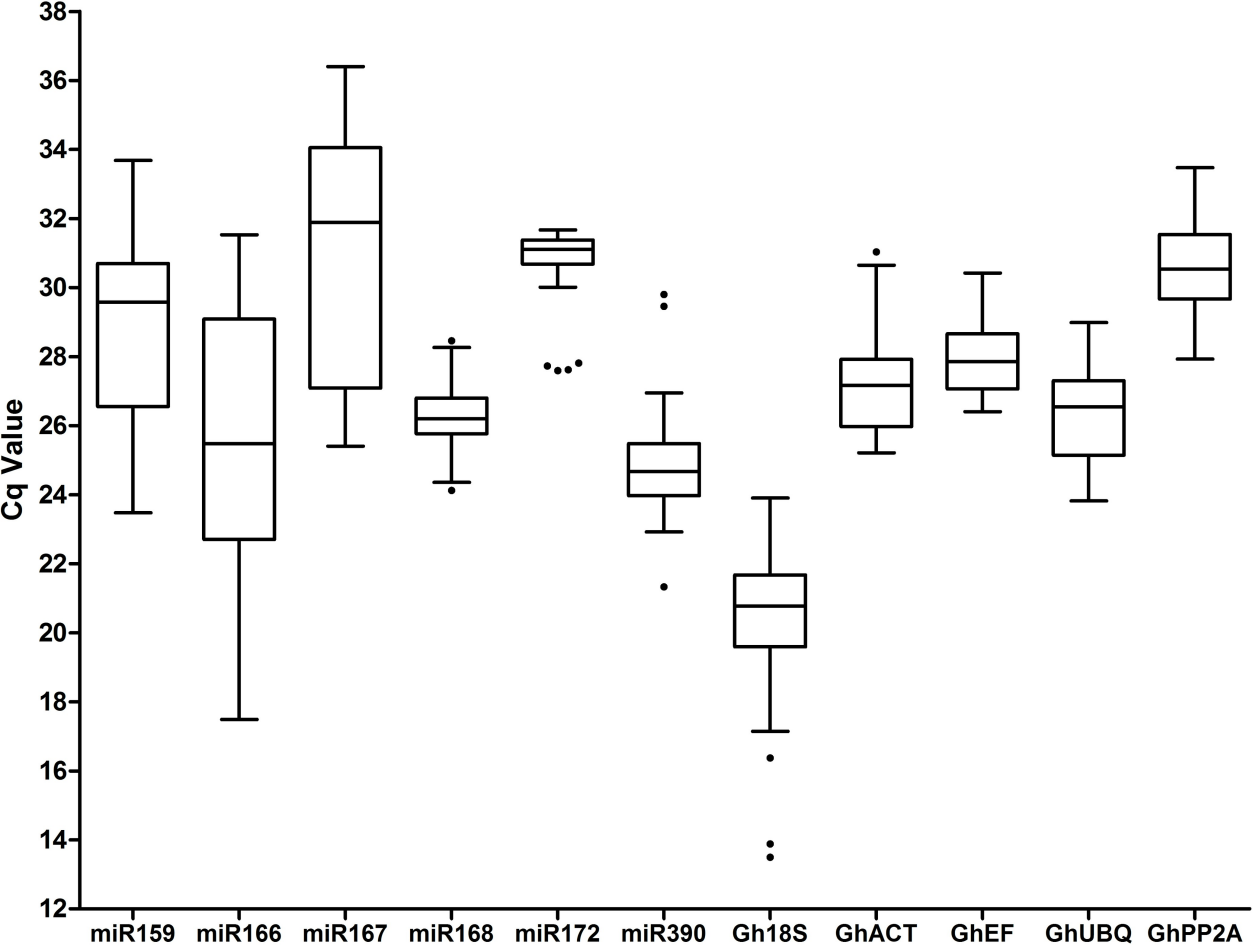
Absolute Cq values of the 11 RG candidates in all samples. Each box indicates the 25/75 percentiles. Whisker caps represent the 10/90 percentiles. The median is depicted by the line, and all outliers are indicated by dots.

To investigate the suitability of 11 candidate genes, we analyzed their expression stabilities based on the 12 different datasets. The transcripts of all the genes were detected using the qPCR technique in all samples. The expression levels displayed a wide range of threshold cycle (Ct) or quantification cycle (Cq) values, with values ranging from 13.50 to 36.40 among the candidates (Fig 1). The *Gh18S* gene was the most highly expressed, with a Cq average of 20.16, while miR167 had a lower expression, with an average Cq value of 31.04. miR168 and miR172 exhibited the lowest variation in the Cq values of all the experimental sets. These results highlight the importance of evaluating the suitable reference genes for gene expression normalization under specific experimental conditions.

### GeNorm, NormFinder, BestKeeper, and ΔCt analysis

To find the best RGs for accurate normalization, it is necessary to determine the most stably expressed gene that has minimal biological variance. Because no unique statistical method exists to approach this issue, we decided to apply the four most frequently used tests to analyze our experimental set: geNorm, NormFinder, BestKeeper and ΔCt.

**Fig 2:**
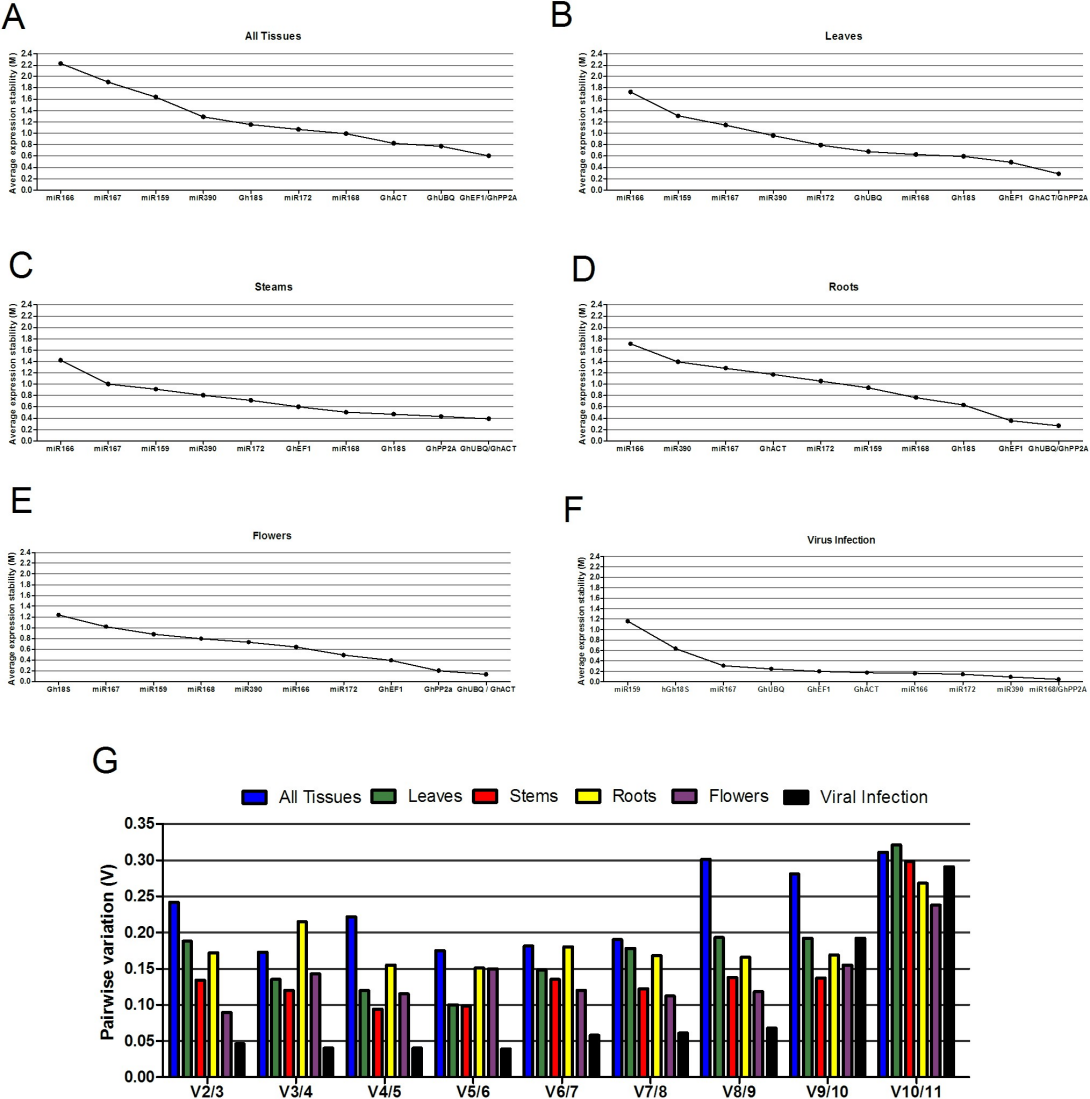
geNorm analysis of the average expression stability values and ranking of the eleven candidate reference genes based on pairwise comparisons. Gene expression stability (M value–y-axis) is shown for the candidate reference genes (x-axis) from the most to the least stable. A- All tissues (set 12); B- leaves (set 1); C—stems (set 2); D—roots (set 3); E- flowers (set 4); and F- virus infection (set 11). G- pairwise variation (V) of the all tissues (set 12), leaves (set 1), steams (set 2), roots (set 3), flowers (set 4) and virus infection (set 11).

The results obtained are summarized in Table 2. For geNorm, the average expression stability (M) value for each gene is calculated based on the average pairwise variation between all genes tested. High gene-expression variability results in high M values and indicates low expression stability. The geNorm results for all the experimental sets analyzed are presented in Fig 2, Table 2 and S2 Table. The lower the M value, the more stable the expression of the reference gene. Values of M that surpassed the cutoff value of 0.15 were not considered stable across the treatments. Thus, according to this algorithm, miR168 and miR390 were the most stable genes, with M scores of 0.049 and 0.096, respectively (Table 2 and S2 Table). In general, the M values for the majority of the other genes were below the cutoff of 0.15, with M scores for a few genes above this value. Curiously, miR166 was a very stable RG with an M value of 0.164 under biotic stress conditions (set 11). However, this miRNA had higher M values in the nine other sets, with an M value that increased to 2.23 for set 12. Once again, this result highlights the need for the careful selection of a good RG for each type of experimental set.

Analyzing the results of all the sets together, we observed that no identical stable reference genes were found for the different experimental sets analyzed in this study. The algorithm indicated that miRNAs were the best RGs for three of the twelve sets studied: miR168/*Gh18S* for leaves and roots (set 6); miR168/miR172 for all organs of DO (set 9); and miR168/*GhPP2A1* for the biotic stress conditions (set 11). For the nine other sets, however, protein-coding genes were found to be the best RGs, with *GhPP2A1*, *GhACT4*, *GhUBQ14*, *Gh18S* or *GhEF1α-5* in the first position depending on the set in the study (Table 2).

The program also includes a pairwise variation (V) implemented as a proxy for the number of genes to be included when normalization is performed using multiple reference genes [41]. Vandesompele and co-workers [42] proposed 0.15 as a cutoff value for V, below which the inclusion of an additional reference gene is not required. Fig 2 summarizes the M and V results for all tissues, leaves, stems, roots and flowers and for the biotic stress condition.

NormFinder considers the intra- and inter-group variations in calculating the normalization factor (NF) and selects genes with the lowest intra-and inter-group variation [43, 49]. In the experimental groups studied, as observed with gGeNorm, distinct reference genes were selected for each experimental set. NormFinder was the method that selected the highest number of miRNAs as RGs. miRNAs were selected as the best RGs in nine sets, with miR172 and miR168 selected six times and three times, respectively. In addition, in eight of the experimental sets, miRNAs were also the second best candidate (Table 2, S2A Fig and S2 Table).

The BestKeeper analysis uses two important criteria for evaluating the best RGs: the stability standard deviation (SD) and its relationship to the BestKeeper index (Pearson correlation coefficient r and p values). The genes that occupied the first position in each set had SD values below 0.5 (Table 2 and S2B Fig). The BestKeeper indicated that miR172 was the best RG in ten of the sets analyzed (1, 2, 3, 5, 6, 7, 8, 9, 10 and 12), meaning that it is a very good RG for gene expression studies in leaves, stem and roots in the individual cultivars and for taking into account all organs together for FM966, DO, and Cedro. For set 11, miR166 was the best RG. For studies in flowers, it would be best to use *GhPP2A1* and miR172 as RGs.

ΔCt is a comparative method that determines the most stable reference genes by comparing the relative expression levels of “pairs of genes” in each tissue sample or treatment and considering the means of the SD values [43]. The ΔCt indicated that miRNAs were the best RGs in seven sets, primarily miR168 (for sets 1, 9, 10 and 12) and miR172 (for sets 4 and 8). For set 11 (biotic stress), miR390 was the best RG, followed by miR172 and miR168 (Table 2 and S2C Fig).

In summary, although no consensus was found among the different methods used to select RGs in the 12 experimental sets tested here, the miRNAs miR172 and miR168 appeared more often as the best RGs, followed by miR390 and the protein-coding genes *GhEF1α-5*, *GhUBQ14* and *GhPP2A1*. With the exception of the geNorm results, the other three algorithms clearly indicated that miRNAs were better RGs than the protein-coding genes, representing at least ¾ of the selected RGs in the 12 sets analyzed.

**Fig 3:**
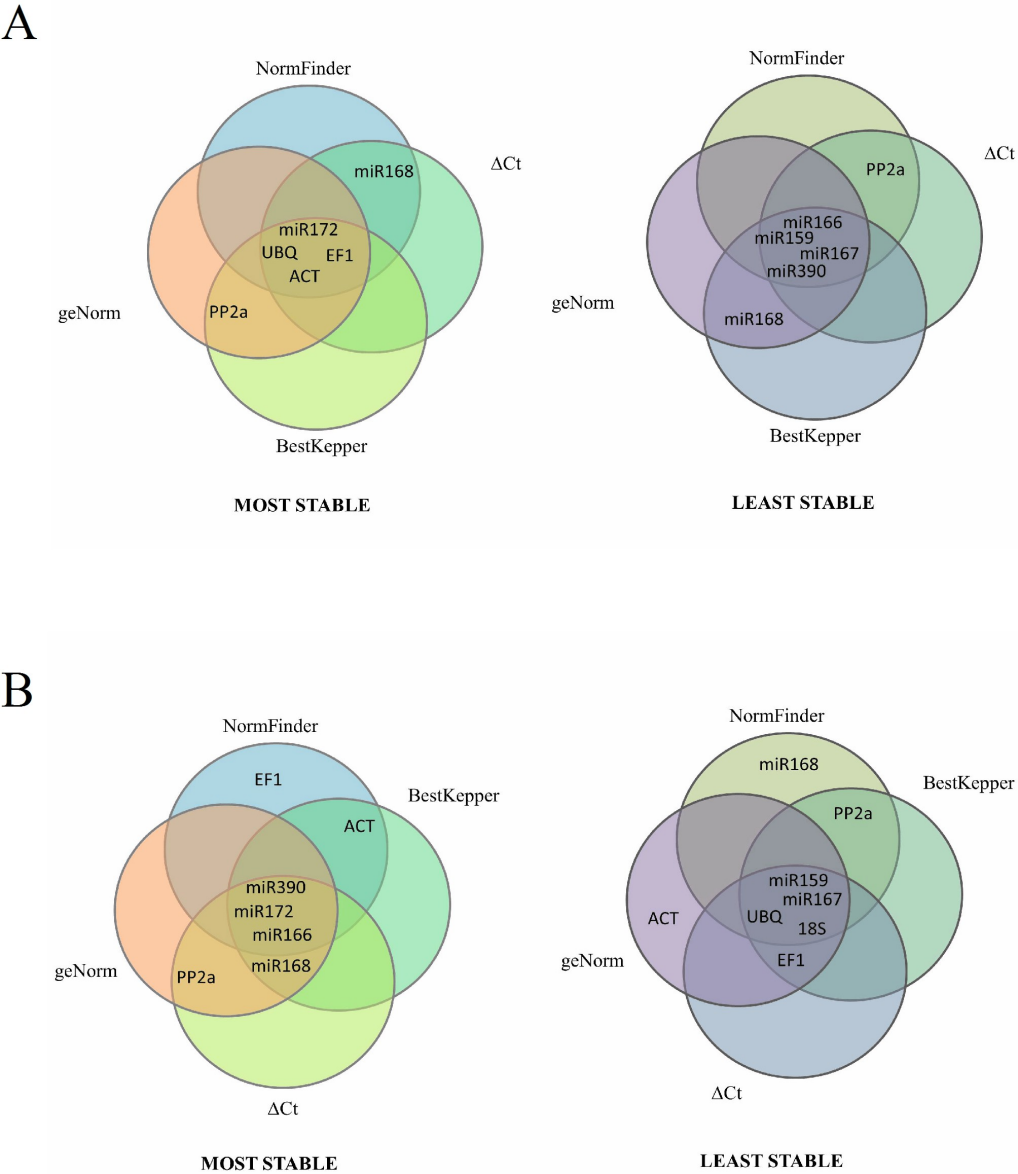
Venn diagram indicating the most and least stable RGs identified by geNorm, NormFinder, BestKeeper and ΔCt. A- Leaves and flowers (set 7). B- Virus infection (set 11).

To provide a clearer illustration of the best RGs selected, Venn diagrams showing the four best RGs selected for leaves and flowers (Fig 3A) and for biotic stress (Fig 3B) are shown.

### Validation of selected reference miRNAs

To validate the selected RGs, three cotton genes coding proteins and six cotton miRNAs for which the expression levels in specific tissues or under stress conditions have been previously studied were chosen. The relative expression levels of *GhMIOX* [46] and *Gh*miR164 [47], in the flowers versus the leaves, and of the cotton *Dicer-like* genes *GhDCL2* and *4* [39] and the miRNAs miR159, miR164, miR2118, miR2910 and miR3476 [37], in virus-infected versus uninfected plants, were assayed.

**Fig 4:**
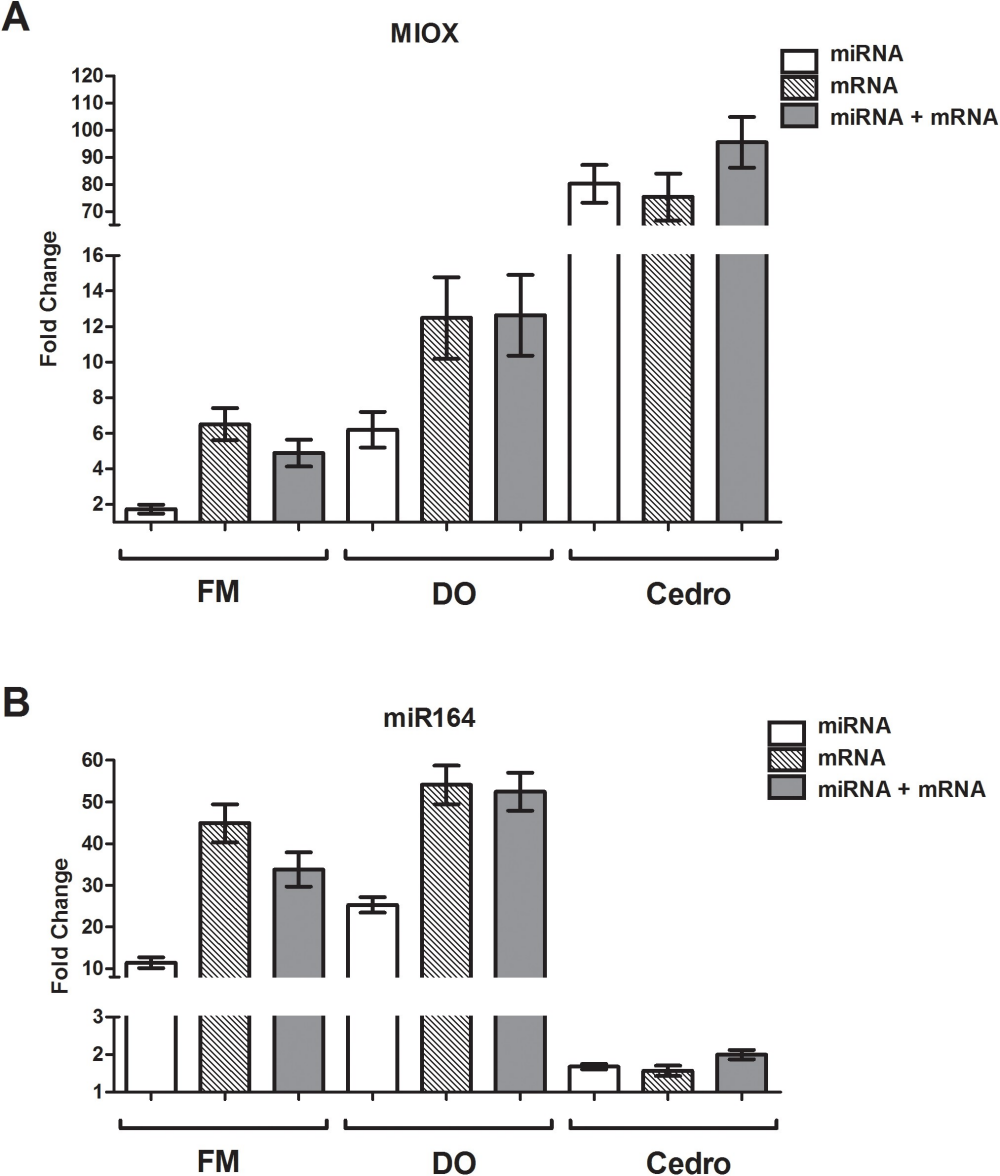
Validation of selected RGs by the relative expression of *Gh-MIOX* and *Gh-*miR164 in flowers of three distinct cotton cultivars [FiberMax966 (FM), Delta Opal (DO), and Cedro]. The relative quantitative values of **A**—*MIOX* and **B**—*miR164* transcripts were obtained by scaling to leaves samples and the normalization was done using reference genes sets of miRNAs: miR172, miR168, and miR390, mRNAs: *GhACT*, *GhPP2A*, and *GhEF*, and miRNA and mRNA: *GhACT*, *GhPP2A*, and miR172. The gene expression was analyzed using the 2^-ΔΔCt^ method and statistical tests were calculated using two-way ANOVA. All the relative expression values showed significant differences (p<0.001) between the expression level in flowers and leaves biological pairs, as determined by Bonferroni method, in exception of the *GhMIOX* transcript in FM (p < 0.05) normalized with miRNAs set.

Studying cotton flower development, Artico and co-workers (2013) observed that *GhMIOX* is highly expressed in flower tissues but is almost undetectable in leaves [46]. In this study, we chose three combinations of RGs in assaying *GhMIOX* expression: a) the use of miRNAs only, b) the use of protein-coding genes only, and c) a combination of miRNAs and protein-coding genes. Based on the geNorm ranking, *miR168*, *miR172* and *miR390* were used in (a); *GhACT4*, *GhPP2A1* and *GhEF1α-5* were used in (b), and *GhACT*, *GhPP2A1* and *miR172* were used in (c). In all three combinations, a higher level of *GhMIOX* expression was observed in flower tissues relative to leaf tissues in all three cotton cvs assayed, as described previously for the Cedro cv (Fig 4A) [46]. It was observed, however, that Cedro appeared to have a higher expression level of *GhMIOX* in the flowers relative to the leaves than the FM966 and DO cvs. In all cases, *GhMIOX* expression patterns were the same, however, highers standard error values were observed for normalization with mRNAs and mRNA plus miRNAs when compared with the standard errors values obtained using miRNAs for normalization, indicating that the use of miRNAs alone seems to results in more accurate normalization in this case.

*miR164* has been shown to have a high expression level in cotton ovules compared to the leaves [47]. Using the same combinations of RG pairs described above for *GhMIOX*, we confirmed the previous observations of Pang and co-workers (2009). *miR164* exhibited an 11–45 times higher expression level in flowers than in leaves (Fig 4B). As expected. depending on the RG combination used, distinct values for relative expression were found, with the highest discrepancy in the relative expression observed when using only miRNAs versus protein-coding RGs or protein-coding plus miRNA RGs (Fig 4). The more stable combinations were achieved using only miRNAs, as can be seen from the standard errors values.

Previous reports have shown that some miRNAs, as well two cotton *Dicer-like* genes, *GhDCL2* and *GhDCL4*, are misregulated during infection by the polerovirus CLRDV [37,39]. Therefore, miR159, miR164, miR2118, miR2910 and miR3476, as well as two cotton protein-coding genes, *GhDCL2* and *GhDCL4*, were assayed for their relative expression levels using the best geNorm RG selected under a condition of biotic stress/CLRDV infection. Based on our reference selection analysis, the use of two reference genes should be sufficient for an accurate analysis under a condition of CLRDV infection because the variation from pair-to-pair (V) was below 0.5. However, we used three reference genes combined as described above. miR168, miR172 and miR390 were used as miRNAs; *GhPP2A1*, *ACT4* and *EF1α-5* were used as protein-coding genes alone, and miR168 and miR390 and *GhPP2A1* were used for the combination.

**Fig 5:**
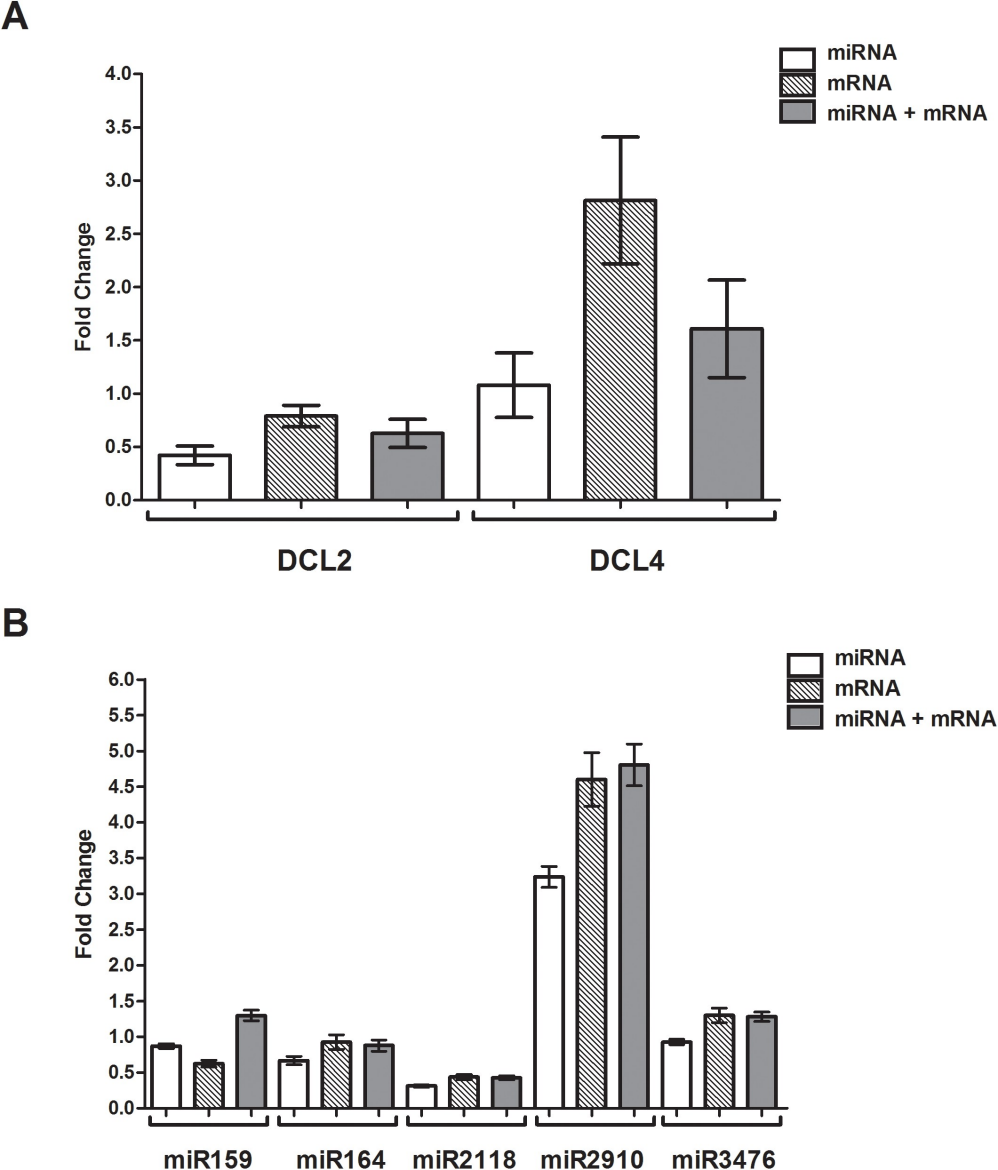
Validation of the RGs by the relative expression of miRNAs and mRNA targets in virally infected (CLRDV) cotton. The relative quantitative values of **A**—cotton Dicer-like (*DCL2* and *DCL4*) and **B—**miR159, miR164, miR2118, miR2910, miR3476 cotton miRNA transcripts were obtained by scaling to uninfected leaves samples and the normalizations were done using reference genes sets of miRNAs: miR172, miR168, and miR390; mRNAs: *GhACT*, *GhPP2A*, and *GhEF*, and miRNA and mRNA: miR168, miR390, and *GhPP2A*. The gene expression was analyzed using the 2^-ΔΔCt^ method and statistical tests were calculated using two-way ANOVA. The significance level between the relative expression values of infected and uninfected leaves, determined by the Bonferroni method, ranged between 000.1>p<0.005 in (A) and (B). No statistical support was obtained for miR2118 normalized with mRNA and with miRNAs and mRNAs.

As previously observed by Silva and collaborators 2011 [39], *GhDCL2* was downregulated (0.42–0.79 fold, depending in the reference genes set used) during CLRDV infection (Fig 5A). *DCL4* showed conflicting results depending on the RG combinations used; the expression values differed among the RG combinations. However, the use of miRNAs as RGs resulted in the lowest standard error values for the two DCL genes assayed (Fig 5A). Reliable differences were observed in the expression levels of miRNAs 2118 and 2910 using the three combinations of RGs (Fig 5B). However, in accordance with the CLRDV cotton infection NGS study [37], the same tendencies of down- and up-regulation were observed. For the other three miRNAs, conflicting results were obtained for each RG combination. The lowest standard error values were observed using miRNAs alone.

## Discussion

qPCR is widely used to investigate gene expression. However, for accurate analysis and interpretation of the results, the choice of suitable genes to normalize the data is a crucial factor. Detailed analyses of various commonly used reference genes, including *ACT*, *GAPDH* and *18S rRNA*, have revealed that they can be significantly influenced by the experimental conditions [35, 50–53]. Therefore, it is necessary to validate reference genes for each plant species being studied and for each specific experimental condition [54]. Reference candidate genes must be evaluated and validated each time a new experimental condition will be analyzed.

Despite cotton agronomic relevance, there is no report concerning reference gene selection for distinct cotton cultivars largely used in the field. Additionally, to date, only four works regarding reference genes for commercial cotton and/or for its model, *G*. *raimondii*, studies have been published [35, 55,56].

To date, only a few studies have reported the use of miRNAs as reference genes for plant gene expression studies [26,28,30,31,32,33]. All these studies, however, revealed that miRNAs are more stably expressed, and as such, are superior RGs compared to protein-coding genes. In this work, we propose the use of miRNAs as RGs for cotton miRNA and protein-coding gene expression profiles. Using 12 experimental condition sets, including different cotton organs from three commercially important cultivars largely planted in Brazil and other important cotton-producing countries (especially in the case of DO) and a biotic stress condition, the CLRDV infection, putative reference miRNAs were compared with five well-characterized protein-coding cotton RGs (*GhACT4*, *GhEF1α-5*, *GhPP2A1*, *GhUBQ14* and *Gh18S*) [35]. The tested Gh-miRNAs (miR159, miR166, miR167, miR168, miR172 and miR390) were representative of distinct functional families.

The algorithms geNorm, NormFinder, BestKeeper and ΔCt were used to select the best reference genes among all the candidates. We observed that even though nearly the same HKGs were selected for the first three positions, the ranking position still varied among the algorithms (Table 2). This discrepancy has already been reported and reflects differences in the calculation of each algorithm [28,30,57–59]. geNorm assesses stability by determining an M value, wherein genes with the lowest M values are those that are more closely correlated; genes with values below 0.15 are considered excellent constitutive genes [42]. However, when the experimental groups analyzed have heterogeneous samples, values between 1.0 and 0.5 are acceptable [60]. Most of the potential HKGs analyzed in this study had M values below 1.0. An application supplied by geNorm provides an indication of the optimal number of reference genes that should be used with a specific experimental condition. As shown by many studies [43,61,62], normalization based on multiple HKGs offers a more accurate assessment, especially when there is no optimal reference gene. In our work, the number of reference genes varied in each experimental set; however, the optimal conditions were increased in most of the sets using only two to three HKGs (sets 1, 2, 4, 5, 8 and 11).

The NormFinder, BestKeeper and ΔCt algorithms all identified miRNAs as the best reference RGs for the majority of the sets. Considering the four algorithms used, miRNAs, especially miR172 and miR168, were the best RGs 32 times out of a total of 48. miR172 was selected as the best candidate 19 times. These results show that the miRNAs used in this study are more stable than the protein-coding genes. Similar results have been reported previously [28,30,31–34], reflecting the importance of selecting miRNAs as RGs for distinct experimental conditions and plants.

miRNA172 has been described in Arabidopsis to negatively regulate three distinct targets: a protein that contains an APETALA2 domain that functions as a transcriptional factor; a subunit of the 26S proteasome, which functions in protein degradation; and a ribonucleoprotein that functions in RNA processing [63,64]. Several studies have shown that miRNA172 has a high expression level in flowers. This had already been observed in cotton and maize and in cotton, miRNA172 expression is a hundred times greater in young flower buds, 0 DPA (days post-anthesis) ovules, and petals than in 2 DPA ovules, stamens and carpels [65,66]. Therefore, miR172 is probably involved in floral development in cotton. Although it has high expression levels in flowers, miR172 was considered a good RG for the study of flower tissues, as we observed in sets 4 and 7. Despite several studies assessing miR172 in the floral tissue of various species, the expression of miR172 in other plant organs has not been deeply studied. Our data suggest that miR172 is an excellent RG for studies in cotton leaves, roots and stems. The second best miRNA, miR168, has no known function in cotton [63], but in Arabidopsis and rice, its target genes are related to signal transduction, development and the stress response [19].

The top two mRNAs identified in our analysis were *GhEF1α-5* and *GhPP2A1*. *Elongation factor 1* has already been described as a stable transcribed gene for tobacco [67] and potato plants [12]. For the cotton cv "Cedro", *GhPP2A1* was better than *GhEF1α-5* for comparing different organs [35]. For biotic stress (Set 11) miRNAs were the best candidates, especially miR390, miR172 and miR168, according to the ranking analysis. miR390 is involved in the development of various plant species [19]. In cotton, miR390 targets protein phosphatase 2C and an ATPase [63]. This miRNA was not defined as a good constitutive reference RG in the other experimental groups. The top mRNAs selected for biotic stress were *GhPP2A1* and *GhACT4*. *PP2A* has been chosen as a RG for qPCR in studying cassava viral infection [68]. Actin has also been described as a reference gene for viral infections in leafhoppers [69].

Using the miRNAs and protein-coding genes selected by geNorm as the best RGs in flowers plus leaves and under biotic stress conditions, we were able to validate the best RG selected. In both cases, miRNAs were the most stable RG, even for the normalization of miRNA and/or protein-coding gene expression, as indicated by their lower standard errors. Artico and collaborators (2010) [35] demonstrated that the promoter *GhMIOX* is 160 times more highly expressed in flowers than in leaves in the Cedro cv using *GhPP2A1* and *GhUBQ14* as RGs. In ours analyses, similar results were obtained for the Cedro cv, while in FM966 and DO a smaller difference was observed. Under a condition of biotic stress, the miRNA candidates had stability values in all the algorithms that were better than those of the mRNA candidates, although the values were above the cutoff value of 0.5 proposed by geNorm [43]. Our analysis showed that each experimental condition tested requires a specific group of reference genes, as observed in many other studies [27,70,71]. This result emphasizes the importance of validating constitutive genes for each experimental condition, particularly when the samples are tissues from different plants, such as coffee [70] and pea [7], for example. In our experiments, *GhUBQ14* and *Gh18S*, both of which are commonly used in cotton qPCR studies, were ranked among the worst RGs under most experimental conditions [72,73].

The present study represents the first effort toward the identification of RG miRNAs in cotton. Combinations of good RG miRNAs were identified in distinct cotton tissues and evaluated in distinct commercially important cvs. Cotton RGs for biotic stress studies are also now available. Additionally, results from the current study demonstrate that miRNAs are more stable and consequently are better reference genes than protein-coding genes for the majority of the sample groups analyzed. Thus, the RGs evaluated here represent important components of future cotton miRNA and protein-coding gene studies.

## Supporting information

S1 Fig **A- qPCR amplification specificity for reference genes**. Dissociation curves of the amplicons for references genes in different organs generated by the qPCR program 7500 Fast Real-Time PCR (Applied Biosystems). The x-axis represents the temperature, while the y-axis indicates the rate of change in the fluorescence of SYBR Green as a function of temperature. **B**- qPCR amplification specificity for target genes. Dissociation curves of the amplicons for the target genes used in this study generated by the qPCR program 7500 Fast Real-Time PCR (Applied Biosystems). The x-axis represents the temperature, and the y-axis indicates the rate of change in the fluorescence of SYBR Green as a function of temperature.
(TIFF)

S2 Fig **A- Stability values generated by NormFinder**. Classification of the expression stability of reference genes for each set analyzed, as generated by the algorithm NormFinder. **B- Average SD of the Cq values generated by BestKeeper**. Classification of the expression stability by averaging the SD of the reference genes analyzed for each set generated by the algorithm BestKeeper. **C**- **Stability values generated by the classification of the expression stability of the candidate genes analyzed for each set generated according to the ΔCt**. **D**—**Stability values (M value) generated by geNorm**. Classification of the expression stability of the candidate genes analyzed for each set generated according to the geNorm. **E**—**Pairwise variation (V) generated by geNorm**. V values identify the optimal number of RGs and V value less than 0.15 indicate that no additional RGs are required to calculate a reliable relative expression. The groups are along the x-axis, and each color represents a certain position that each gene occupies, ranging from the first position (most stable) to the eleventh position (less stable). On the y-axis, their stability values for each position are indicated. All results summarized in the S2 Table.
(TIF)

S1 Table List of oligonucleotides used in the qPCR assays for the validation of RGs. The amplicon sizes and primer sequences used for the detection of the Gh-miRNAs and mRNAs using real-time quantitative PCR.
(DOC)

S2 Table Stability values for each experimental set and analysis of all candidate genes in the four methods studied. (XLSX)
