# Supplementary figures and images for: microRNAs as reference genes for quantitative PCR in cotton
Source: PLoS One. 2017 Apr 17;12(4):e0174722. doi: 10.1371/journal.pone.0174722 (PMC5393557; doi:10.1371/journal.pone.0174722)

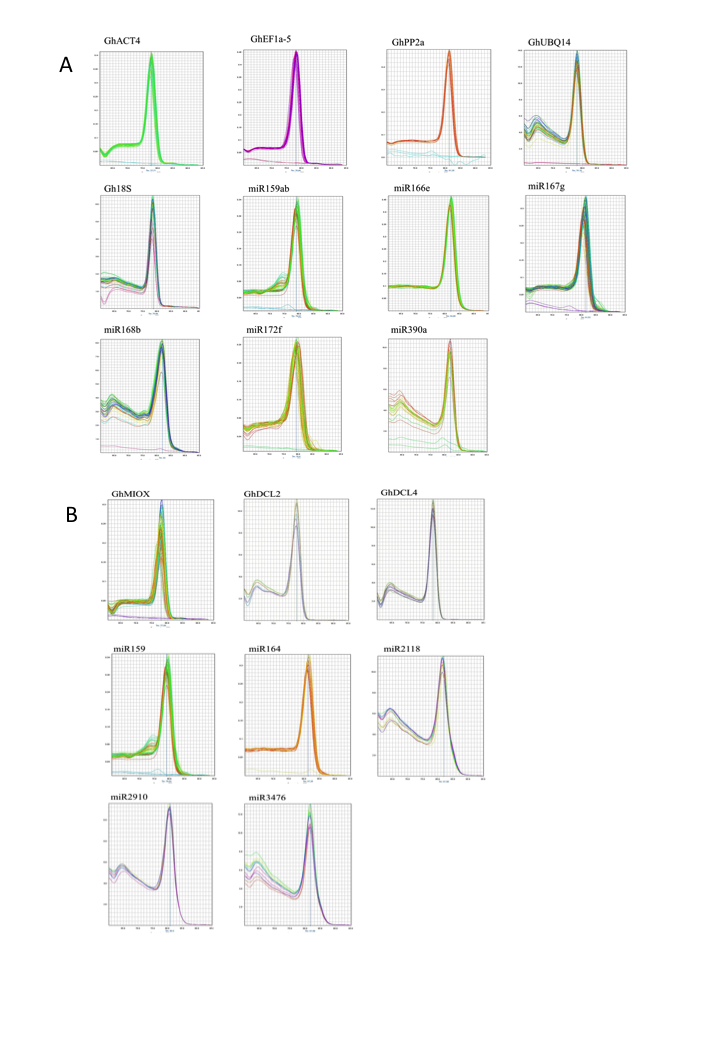

Supplement: S1 Fig — A- qPCR amplification specificity for reference genes. Dissociation curves of the amplicons for references genes in different organs generated by the qPCR program 7500 Fast Real-Time PCR (Applied Biosystems). The x-axis represents the temperature, while the y-axis indicates the rate of change in the fluorescence of SYBR Green as a function of temperature. B- qPCR amplification specificity for target genes. Dissociation curves of the amplicons for the target genes used in this study generated by the qPCR program 7500 Fast Real-Time PCR (Applied Biosystems). The x-axis represents the temperature, and the y-axis indicates the rate of change in the fluorescence of SYBR Green as a function of temperature. (TIFF) [file pone.0174722.s001.tiff]

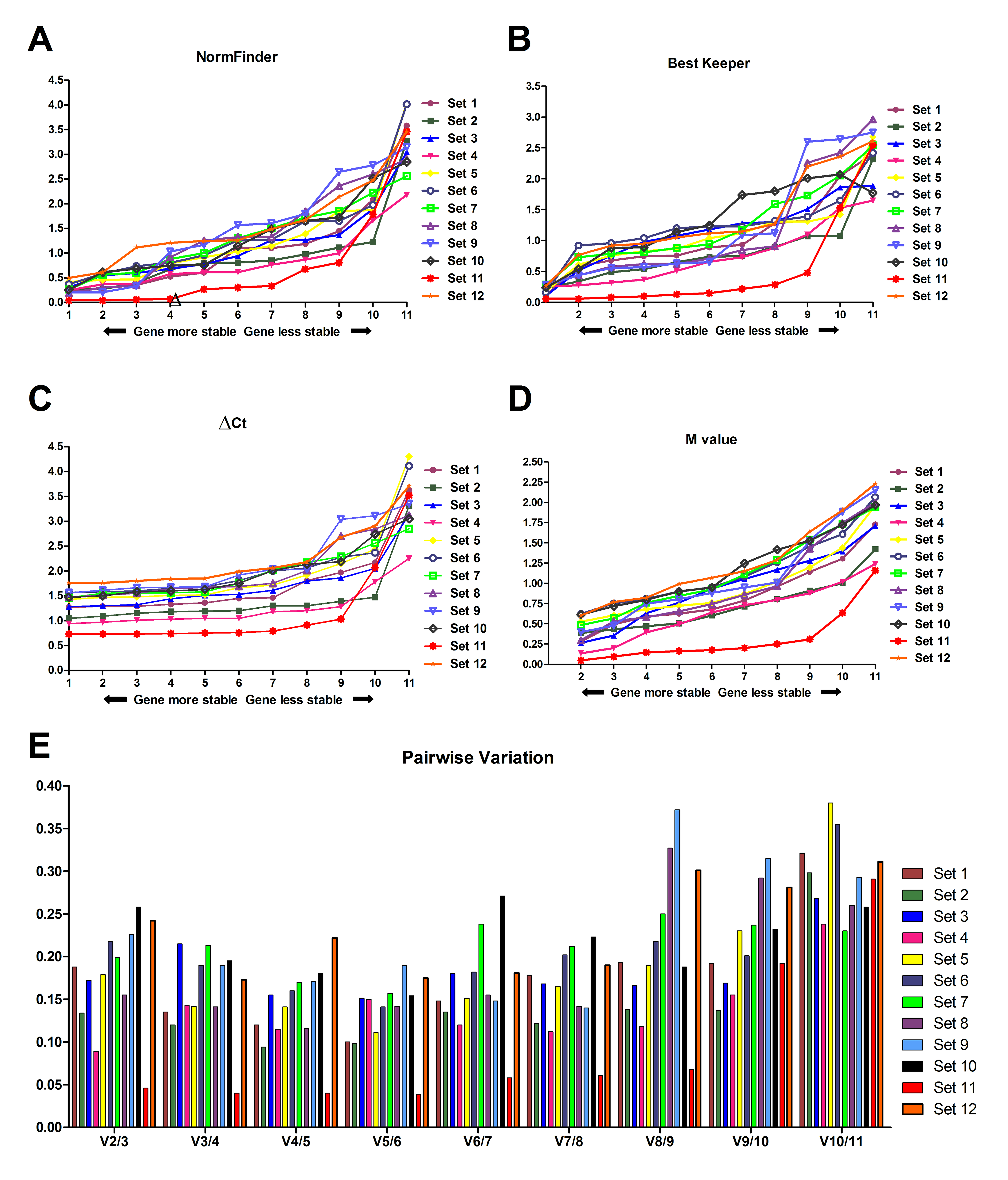

Supplement: S2 Fig — A- Stability values generated by NormFinder. Classification of the expression stability of reference genes for each set analyzed, as generated by the algorithm NormFinder. B- Average SD of the Cq values generated by BestKeeper. Classification of the expression stability by averaging the SD of the reference genes analyzed for each set generated by the algorithm BestKeeper. C- Stability values generated by the classification of the expression stability of the candidate genes analyzed for each set generated according to the ΔCt. D—Stability values (M value) generated by geNorm. Classification of the expression stability of the candidate genes analyzed for each set generated according to the geNorm. E—Pairwise variation (V) generated by geNorm. V values identify the optimal number of RGs and V value less than 0.15 indicate that no additional RGs are required to calculate a reliable relative expression. The groups are along the x-axis, and each color represents a certain position that each gene occupies, ranging from the first position (most stable) to the eleventh position (less stable). On the y-axis, their stability values for each position are indicated. All results summarized in the S2 Table. (TIF) [file pone.0174722.s002.tif]
