# Supplementary material for: microRNAs as reference genes for quantitative PCR in cotton
Source: PLoS One. 2017 Apr 17;12(4):e0174722. doi: 10.1371/journal.pone.0174722 (PMC5393557; doi:10.1371/journal.pone.0174722)
Supplement: S1 Table — The amplicon sizes and primer sequences used for the detection of the Gh-miRNAs and mRNAs using real-time quantitative PCR. (DOC) [file pone.0174722.s003.doc]

**Supplemental table 1: List of oligonucleotides used in qPCR assays for the validation of RGs.** The amplicon size and primers sequences used for the detection of Gh-miRNAs and mRNA using real-time quantitative PCR.

| Gene abbreviation | Symbol | Primer names | Primers sequences (5’-3’) | Amplicon (bp) | Reference |
| --- | --- | --- | --- | --- | --- |
| GhMIOX | MIOX | GhMIOXF | TGAAGGATGTGGACTGGACA | 147 | [49] |
|  |  | GhMIOXR | CCATGTTTGTGCAATGGGTA |  |  |
| GhDCL2 | DCL2 | GhDCL2F | GATCGCTATCATGCTTCTCCGCAG | 81 | [44] |
|  |  | GhDCL2R | TGGGGAACCAAGAAGACAGCGAA |  |  |
| GhDCL4 | DCL4 | GhDCL4F | GCTTCCAAGCGGCAACAGCATT | 186 | [44] |
|  |  | GhDCL4R | AGGATGCACAATCGCCTGAAGGAG |  |  |
| miR159* | miR159 | miR159F | GCGGCGGTTTGGATTGAAGGGA | 72 | [38] |
|  |  | miR159R | GTTGGCTCTGGTGCAGGGTCCGAGGTATTCGCACCAGAGCCAACCCAGAGC |  |  |
| miR164* | miR164 | miR164F | GCGGCGGGTGGAGAAGCAGGGCA | 72 | [38] |
|  |  | miR164R* | GTTGGCTCTGGTGCAGGGTCCGAGGTATTCGCACCAGAGCCAAC CTGCACG |  |  |
| miR2118* | miR2118 | miR2118F | GCGGCGGGCCGATTCCACCCA | 72 | [38] |
|  |  | miR2118R | GTTGGCTCTGGTGCAGGGTCCGAGGTATTCGCACCAGAGCCAACCTAGGCA |  |  |
| miR2910* | miR2910 | miR2910F | GCGGCGGTAGTTGGTGGAGCGA | 72 | [38] |
|  |  | miR2910R* | GTTGGCTCTGGTGCAGGGTCCGAGGTATTCGCACCAGAGCCAACCGACAAA |  |  |
| miR3476* | miR3476 | miR3476F | GCGGCGGTGAACTGGGTTTGTT | 72 | [38] |
|  |  | miR3476R | GTTGGCTCTGGTGCAGGGTCCGAGGTATTCGCACCAGAGCCAACCGCAGCC |  |  |

* - stem-loop RT primers were used for miRNA amplification as Chen et al. 2005 [20]. The underlined nucleotides was specific to the miRNA target.

**Supplementary table 1 from manuscript**

**miRNAs as housekeeping genes for gene expression studies in cotton**

Authors: Anna Karoline Silva Fausto^1^, Tatiane Silva^1, 2^, Elisson Romanel^1, 2^, Maite F S Vaslin^1*^

^1^Lab. Virologia Molecular Vegetal, Depto. Virologia, IMPPG, Universidade Federal do Rio de Janeiro, UFRJ, Rio de Janeiro, RJ, Brasil

^2^Departamento de Biotecnologia, Escola de Engenharia de Lorena (EEL), Universidade de São Paulo (USP), 12602-810, Lorena, SP, Brasil

Corresponding author: Maite F S Vaslin, [maite@micro.ufrj.br](mailto:maite@micro.ufrj.br), +55212560-8028, fax +552125608344.
